# How do short videos influence users’ tourism intention? A study of key factors

**DOI:** 10.3389/fpsyg.2022.1036570

**Published:** 2023-01-17

**Authors:** Jing Liu, Yujie Wang, Liyan Chang

**Affiliations:** ^1^School of Management, Nanjing University of Posts and Telecommunications, Nanjing, China; ^2^School of Information Management, Nanjing University, Nanjing, China

**Keywords:** short video, tourism intention, SOR model, technology acceptance model, flow theory factor structure relevant studies

## Abstract

**Background:**

Short videos play a key role in the process of tourism destination promotion, and attractive short videos can bring tourist flow and economic income growth to tourist attractions. Many tourist attractions in China have achieved remarkable success through short video promotion.

**Purpose:**

The purpose of this study was to investigate the behavioral characteristics of short video users browsing short tourism videos and explore what factors of short video affected users’ tourism intention. This study also compared which factors were most important in triggering users’ tourism intention in marketing communication *via* short tourism videos in order to shed light on tourism destination strategy and facilitate adaptation to market development trends.

**Methods:**

This study developed a conceptual model by extending the stimulus-organism-response (SOR) model with technology acceptance factors (perceived usefulness, perceived ease of use) and short video factors (perceived enjoyment, perceived professionalism, perceived interactivity) to examine users’ tourism intention. A convenience random sampling technique was used to distribute the questionnaire in Chinese city of Nanjing. Four hundred twenty-one respondents participated in the questionnaire, with 395 providing valid data.

**Results:**

The results of the SEM analysis show that all posed hypotheses (Perceived professionalism - > Telepresence, Perceived interactivity - > Telepresence, Perceived enjoyment - > Telepresence, Perceived ease of use - > Telepresence, Perceived enjoyment - > Flow experience, Perceived ease of use - > Flow experience, Telepresence - > Flow experience, Telepresence - > Tourism intention, Flow experience - > Tourism intention) are confirmed except for (Perceived usefulness - > Tourism intention), which is not confirmed.

**Conclusion:**

The findings of this study will help fill the gap in previous research on the relationship between short video influencing factors and users’ tourism intention, thus contributing to the academic research on emerging short videos and the endorsement of destinations promoted by technological innovation.

## 1. Introduction

Short video platforms are a relatively new global phenomenon with a rapidly increasing number of users (Xie et al., 2019). Consumers and brands have become more inclined to communicate with each other through short video platforms (Bailey et al., 2021; Kim et al., 2021; Wajid et al., 2021). In China, under the leadership of TikTok, Kwai and other platforms, short videos have quickly attracted people of all ages (Du et al., 2022). Unlike mini movies or most YouTube videos, short videos are usually only 30 s to 1 min long, and their short duration fits in with today’s fast-paced life. Their rich and interesting background music has effectively enhanced users’ social ability (Cao et al., 2021).

High-quality short tourism videos display the natural scenery and cultural highlights of tourist attractions through short video platforms such as TikTok (Wei and Tong, 2019), and package the whole in a form suitable for dissemination, attracting tourists to visit these attractions (Chen et al., 2022). The precedent of successful tourism short video marketing, as in the case of the Chinese city of Chongqing (Liu and Fu, 2021), has led more and more researchers to explore the impact of tourism destination short video factors on tourism intentions (Cao et al., 2021; Li, 2021). However, the process of information search and consumption in the tourism field is complex, and more research is needed on how short videos affect tourism intentions (Pesonen and Pasanen, 2017; Alamäki et al., 2019).

From the perspective of tourism destinations and short video creators, it is important to understand how to develop video content based on potential consumers and what factors make the media effective (Yang et al., 2022). After investigating TikTok, some scholars found that most of the short tourism videos on the platform lack interesting and professional content, and the tourism intention of users is rarely improved as a result (Alamäki et al., 2019). Wang et al. (2022) used the extended Technology Acceptance Model (TAM) to discuss the impact of perceived playfulness of short video on theme park tourism intentions. Although previous research verified the positive effect of short videos on potential tourists’ travel intention (Shani et al., 2010; Rafael et al., 2020; Wengel et al., 2022), there is a lack of research on the main factors by which such videos affect tourism intention. Short video producers need to consider the information they want to convey and how different types of people will handle it (Watzlawick et al., 1967).

In this study, we investigated the basic information about the population, content and habits involved in browsing short tourism videos, as well as the impact of the technology and content of short videos on tourism intention (Zhang et al., 2019; Wang, 2020; Yan, 2021). We construct an influencing factor model to describe how short videos affect users’ tourism intention based on the extended SOR model, to which we add factors of perceived enjoyment (PE), perceived professionalism (PP), perceived interactivity (PI), perceived usefulness (PU) and perceived ease of use (PEOU). Although these factors are considered the potential key factors for adoption in many fields, they have never been tested in empirical research on the use of the SOR model, nor have they been tested in research on tourism intention. Therefore, this study aims to investigate the impact of these short video factors (PE, PP, PI, PU, PEOU) on users’ tourism intention. To this end, the core contribution of this study has two aspects:

- To develop the conceptual model by extending the SOR model with external factors related to short video and investigate the impact of short video on tourism intention.
- To investigate the proposed study framework using AMOS 26.0 and structural equation modeling (SEM).

The rest of this study is organized as follows: Section 2 provides a literature review through the lenses of short videos, tourism intention, and the SOR model. Section 3 tackles hypothesis development and the research model. Section 4 presents the research methodology, and Section 5 shows the results, which are discussed in Section 6. Section 7 provides the theoretical and practical implications. Section 8 demonstrates the limitations and offers future recommendations, and Section 9 concludes the study.

## 2. Literature review

### 2.1. Short video research

The rise of short video relies on the emergence of short video applications. The earliest short video application, Viddy, was founded in the United States in 2010 and officially released on April 11, 2011. This platform made it convenient for users to create and share videos. In 2015, some researchers defined short video as a video shot by a mobile device for rapid editing or beautification and social sharing, with a duration of 5–15 s (Wang et al., 2015). Later, the *Research Report on the Development of Short Video Industry* (Fu, 2019) suggested a broader duration of 5 min or less, defining short videos as those characterized by short duration, fast dissemination speed, low creation threshold and strong participation. Others have described short video as a new form of expression combining words and images. Its emergence benefits from the reduction of network fees, the acceleration of network speed and the popularity of various intelligent devices. Users can often search for like-minded others, socialize, learn, and express themselves through such media (Li, 2018; Fu, 2019; Kim et al., 2021). Although researchers have different definitions of short video, they all include the following points: (1) a certain time limit, usually within 5 min and most often 15 s to 1 min; (2) simple production and editing processes that yield vivid, impressive content; (3) convenient dissemination and sharing, mainly on social media; and (4) meeting individual needs and resonating in the minds of viewers.

Compared with long videos, short videos have low dissemination cost and high efficiency, especially in meeting the entertainment needs of contemporary young people’s fragmented lifestyles. As the main consumers of tourism, the post-90s and post-00s are bound to bring about changes in the industry’s mode of dissemination (Buhalis, 2000). Among those effects brought about by short video dissemination, the promotion of urban destinations is particularly prominent. Chongqing, Chengdu, Nanning and other cities have become “Internet-popular” cities through the promotion of short video platforms in 2021, objectively reshaping the cities’ image and promoting the dissemination of urban brand culture. However, the “virality” of urban culture also exposed the problems of lower popularity for content related to historical, cultural and natural/scenic attractions, along with platform homogenization and poor supervision within the short video ecosystem (Li and Wang, 2019). Therefore, some researchers have made bold innovations in the originality, production and audio-visual language presentation of videos. They selected benchmark characters and cultural landmarks and integrated emotion, information and artistry into content creation, so as to explore the feasibility of recording and disseminating urban culture through short videos in the future (Chen, 2018).

In the mobile Internet era, tourism short videos have gradually become an important channel for people to pursue freshness and satisfy curiosity. They are a convenient way for online potential tourists to find information about a destination (Karpinska-Krakowiak and Eisend, 2019). Combining previous studies with the specific characteristics of tourism video content, we defined tourism short videos as short videos of local cuisine, urban or natural landscapes, or commercial or historical tourist attractions, uploaded or shared by the local government, enterprises, tourists and local residents, with the short video platform as the dissemination carrier. Recently, Zhang and Hu (2021) have also summarized three types of value characteristics of such videos, namely: the sensory experience creates a refreshing audio-visual feast, a large number of Internet content creators bring the diversity and richness of the tourism experience, and social interaction forms a more profound impression of the tourism destination.

### 2.2. Tourism intention research

The concept of tourism intention first evolved from consumers’ purchase intention. Gartner (1994) posited that tourism intention is tourists’ attitude toward a tourism destination. Chen and Tsai (2007) put forward that tourism intention includes two meanings: recommendation intention and revisit intention, which is the judgment of tourists on the possibility of revisiting a tourism destination or recommending that destination to others. Huang and Huang (2009) pointed out that tourism intention and motivation are closely related. Tourism motivation can drive tourism intention, which leads tourists to choose a given destination and generates purchase behavior.

Researchers mainly study tourism intention from four aspects: perceived value to tourists, tourism destination, environmental factors and social media. Under COVID-19, the research direction gradually turned to the factors influencing digital tourism intention. Influencing factors in cultural tourism intention have also drawn recent attention from researchers (Miao, 2020; Bieszk-Stolorz et al., 2021). The Stimulus-Organism-Response (SOR) model, first proposed by Woodworth in 1929, was initially used to explain and analyze the impact of environment on human behavior, then gradually developed into a basic model to study consumer behavior (Woodworth, 1929). The model has been widely used in the research on tourism behavior intention and tourism behavior. He et al. (2021) found that when the tourism destination has a good reputation and high perceived service quality, tourists will have higher satisfaction and stronger environmental responsibility behavior. Based on the SOR model, Wang (2021) verified that the reference information of educational excursions has a significant influence on educational excursion intention by constructing the relationship model among subjective knowledge, reference information, perceived risk, perceived value and tourism intention. Table 1 summarizes the factors found to have a significant impact on tourism intention in some existing studies.

**Table 1:** Constructs and their sources.

Constructs	Sources
Perceived enjoyment	Wilder et al. (1989), Beuckels and Hudders (2016), Xue (2017), Choi et al., 2021
Perceived professionalism	Zeng and Lu (2021), Li et al. (2022), Ma and Zhang (2022)
Perceived interactivity	Moon and Kim (2001), Thorson and Rodgers (2006), Zhao and Lu (2010), Wu et al. (2014), Choi et al. (2021), Zhou et al. (2022)
Perceived usefulness	Lee and Lehto (2013), Hansen et al. (2018), Yahia et al. (2018), Yan (2021), Hyowon et al. (2022), Wang et al. (2022)
Perceived ease of use	Hansen et al. (2018), Yahia et al. (2018), Yan (2021), Hyowon et al. (2022), Wang et al. (2022)
Telepresence	Minsky (1980), Lombard and Jones (2007), Pelet et al. (2017), Kim and Ko (2019), Yao and Jia (2021)
Flow experience	Csikszentmihalyi (1975), Koufaris (2002), Valenzuela et al. (2009), Gong et al. (2019), Ilhami (2021)

Many researchers have studied tourism intention from the personal perspective (tourism motivation, tourism attitude, self-efficacy, mental imagery, flow experience) and the environmental perspective (destination image, subjective norms, telepresence, perceived risk). At present, there are relatively few studies on the influence of short-video-related factors on tourism intention. As a new media form, short videos have great advantages in recording real performance of events and highlighting beautiful scenery, but it is unknown whether they can attract tourists. In addition, perceived ease of use (PEOU) and perceived usefulness (PU) from the TAM are often used as prerequisites for the study of behavioral intention. By combining the content characteristics of short videos with the theoretical factors of technology acceptance, this paper constructs an extended SOR model to explain and predict users’ tourism intention.

### 2.3. Stimulus-organism-response model and technology acceptance model

Mehrabian and Russell (1974) put forward the stimulus-organism-response (SOR) theory. Compared with the “stimulus–response” theory in behavioral psychology, the SOR theory pays more attention to the analysis and interpretation of the psychological activity process of the organism, systematically explains what psychological factors are responsible for the occurrence of individual behavior, and effectively clarifies the mechanism of influence between stimulus and individual behavior intention (Donovan and Rossiter, 1982). In the context of cognitive learning the (SOR) model defines stimulus (S) as a factor that affects individual cognition or emotional activities. Organism (O) is an individual’s psychological or cognitive state formed by stimulus factors, while response (R) is an individual’s behavioral response realized through emotional and cognitive processes (Donovan and Rossiter, 1982). SOR is suitable for studying consumer behavior intention because it focuses on people’s internal emotions and cognitive factors (Sherman et al., 1997; Walsh et al., 2011; Tian and Lee, 2022).

Although the SOR theory was proposed prior to the advent of the Internet for offline behavior, it is now widely used in research into online user behavior. For example, Chen and Yao (2018) verified the impact of website framework quality on consumers’ impulse buying behavior in mobile auctions; Luqman et al. (2017) studied how transitional social use, transitional cognitive use and transitional hedonism caused users’ sense of technical stress and fatigue, which led to users’ voluntary abandonment of social networking sites. In addition, the SOR model proposes an internal mechanism, with both emotional and cognitive components, for factors influencing online user behavior, which allows for exploration of the internal psychological changes of consumers in more detail and improves the relevant research on consumer behavior. Tian and Lee (2022) proposed a study on the impact of social e-commerce fashion products on continuous purchase intention, and explored the relationship between social media interactivity, perceived value, immersive experience and continuous purchase intention. Based on the SOR model, other scholars have analyzed the impact of doctor information on patients’ cognitive trust and emotional trust, along with the impact of patient trust on doctors’ choice behavior, by using eye tracking technology and questionnaire survey methods, and found that emotional trust triggered by doctor information has a greater impact on patients’ choice behavior (Shan et al., 2019). Others used the SOR model to analyze how the richness of social business characteristics affected consumer perception, cognitive factors and emotional factors, and how it affected consumer website stickiness (Friedrich et al., 2019).

In keeping with previous studies, we choose the SOR model to explore the impact of short videos on tourism intention, using short videos factors as stimulus variables, telepresence and flow experience as organism variables, and tourism intention as the response variable. The research model for this study was the extended SOR model with the addition of the technology acceptance factors and short video variables.

Davis (1989) put forward the TAM in 1989 on the basis of rational behavior theory (Davis et al., 1989). The main purpose of this model is to explain and predict users’ technology use behavior by studying the influencing factors of people’s acceptance and use of new technologies. The model believes that behavior intention generates use behavior, and behavior intention depends on individual perceived usefulness and behavior attitude. Among them, behavioral attitude is determined by perceived ease of use and perceived usefulness (Davis, 1989).

In this paper, perceived usefulness (PU) and perceived ease of use (PEOU) in the TAM model are used as perceptual variables to measure the flow experience and telepresence of users when watching tourism short videos, so as to judge the impact on travel intention.

### 2.4. Research hypotheses

**Figure 1:**
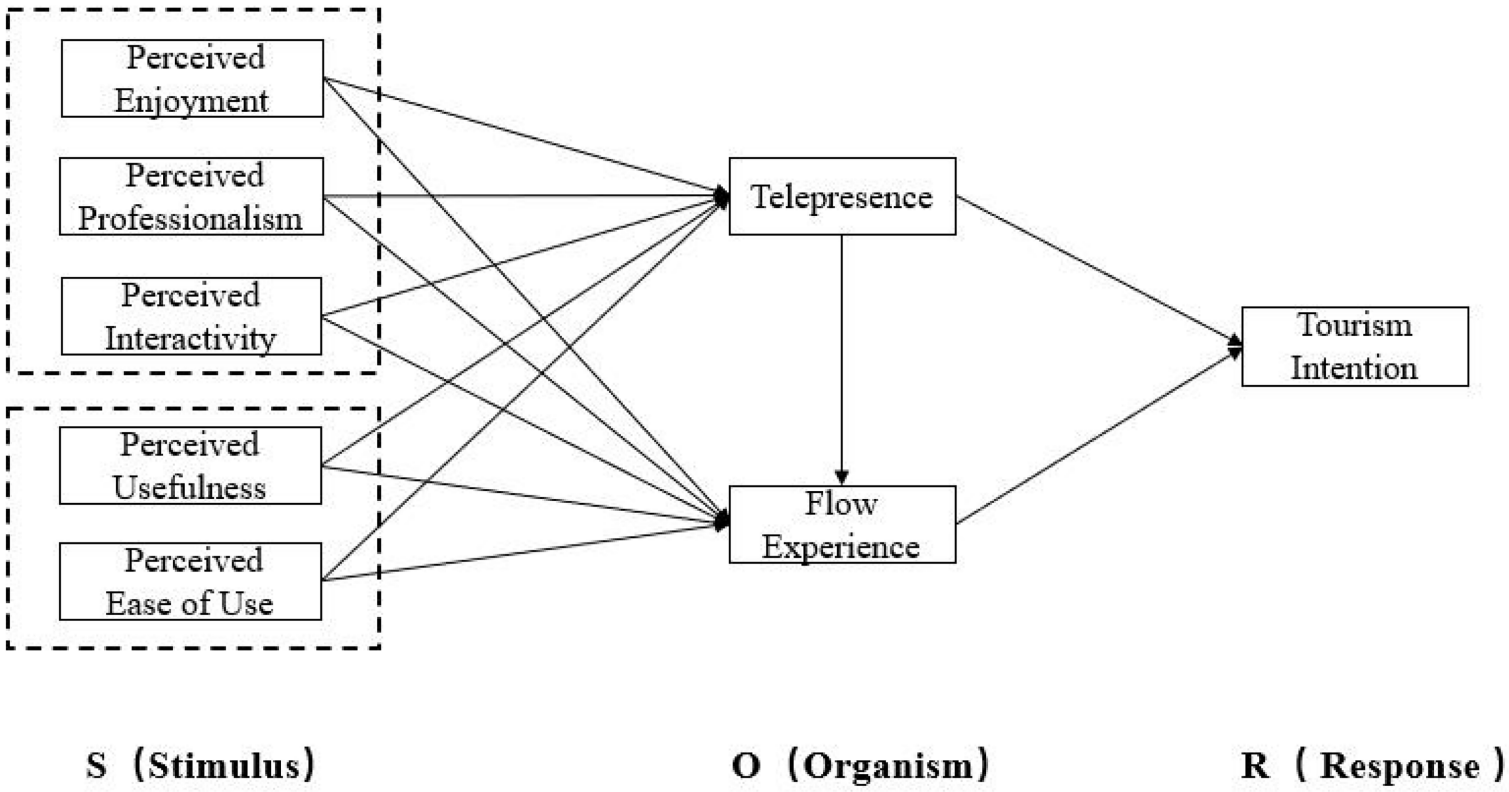
Research model of users’ tourism intention.

Based on TAM and flow theory, this paper extends the SOR model by using the characteristic variables of short videos and constructs a research model of users’ tourism intention to verify the impact of perceived enjoyment (PE), perceived professionalism (PP), perceived usefulness (PU), perceived interactivity (PI), perceived ease of use (PEOU), telepresence (TP) and flow experience (FL) on users’ tourism intention (TI). The research model is shown in Figure 1.

According to the tourism intention model, the following hypotheses are:

*H1a*: Telepresence (TP) has a positive impact on users’ travel intention (TI).

*H1b*: Telepresence (TP) has a positive impact on flow experience (FL).

*H2*: Flow experience (FL) has a positive impact on users’ tourism intention (TI).

*H3a*: Perceived enjoyment (PE) has a positive impact on telepresence (TP).

*H3b*: Perceived enjoyment (PE) has a positive impact on flow experience (FL).

*H4a*: Perceived professionalism (PP) has a positive impact on telepresence (TP).

*H4b*: Perceived professionalism (PP) has a positive impact on flow experience (FL).

*H5a*: Perceived interactivity (PI) has a positive impact on telepresence (TP).

*H5b*: Perceived interactivity (PI) has a positive impact on flow experience (FL).

*H6a*: Perceived usefulness (PU) has a positive impact on telepresence (TP).

*H6b*: Perceived usefulness (PU) has a positive impact on flow experience (FL).

*H7a*: Perceived ease of use (PEOU) has a positive impact on telepresence (TP).

*H7b*: Perceived ease of use (PEOU) has a positive impact on flow experience (FL).

## 3. Methodology

### 3.1. Participants

The participants in this study are short video lovers—defined here as people of any age who often browse various new media platforms (TikTok, YouTube, etc.). Short video lovers are divided into two main categories: creators of original video content and the audience watching the videos. It has been found that the vast majority of users believe that they have the freedom to choose short video apps in terms of time, place and content—including relevance to personal interests and hobbies (Chen and Chen, 2022). Therefore, users who often browse short videos may become the audience of tourism short videos.

The participants were required to meet the following criteria: (1) they were short video lovers who often browsed short video and (2) they volunteered for the study. We used the convenience sampling technique in collecting the primary data, which were from the Chinese city of Nanjing. Boomsma (1987) has suggested that, when using the maximum likelihood method to estimate the structural equation model, the sample size be 5–10 times the number of questionnaire items, with a minimum sample size of 200. To ensure adequate age coverage, the age of short video lovers is segmented as follows: under 18 years old, 18–30 years old, 30–55 years old and over 55 years old. In total, 421 short video lovers participated in this study, of which 395 participants’ data was deemed valid after eliminating those with obvious filling errors (e.g., identical answers from beginning to end, or too many missing values in the questionnaire). These respondents included 186 men and 209 women, for a nearly 1:1 gender ratio.

### 3.2. Instrument

In order to explore the influencing factors of short tourism videos on users’ tourism intention, this paper conducts a questionnaire survey of people who have a habit of browsing short videos. Based on previous studies, the authors understood the possible initial measurement items, conducted small-scale interviews with short tourism video lovers and professionals in relevant research fields, and then designed a questionnaire. The questionnaire partly adopted a Chinese translation of the scale from previous study, with some new items designed according to the interview results. The resulting closed questionnaire includes an item on “whether you have watched short tourism videos,” so as to eliminate invalid responses. To ensure the quality of the questionnaire, a small-scale pre-survey was carried out with 30 college students who often browse short videos, and the reliability and validity of the questionnaire were analyzed. In combination with the opinions of five professionals in the field, some measurement items involved in the questionnaire were modified to produce a formal questionnaire.

The questionnaire is divided into two parts, the first of which collects users’ basic information: gender, age, interest in tourism and viewing situation of short tourism videos. The second part surveys users’ tourism intention after browsing short tourism videos. The indicators representing the constructs were adopted from previous studies (Table 1). The questionnaire uses a Likert 5-point scale with values ranging from “strongly disagree” to “strongly agree.”

### 3.3. Data collection

The data was collected using a third-party online survey software “questionnaire star” (www.wjx.cn), which was unconnected to any institutional system from which the research samples were collected. In order to protect the confidentiality and anonymity of the respondents, the questionnaire data did not collect the names, email addresses or phone numbers of the respondents. In addition, demographic data are collected by grade and subject.

Before data collection, the School of Management of Nanjing University of Posts and Telecommunications issued an official license for the project, so as to issue large-scale online questionnaires. Before filling in the questionnaire, participants were informed of the survey intention, and the meaning and content of the tourism short videos were explained, so as to ensure the respondents’ understanding of the questionnaire and the authenticity of the data in reflecting users’ tourism intention. The appraisers of the project shall collect the questionnaire from April 26 to May 3, 2022 and complete the screening inspection in time. It should also be mentioned that participants are told that if they do not want to fill out the questionnaire, they can ask to exclude themselves. Finally, the participants got the contact information of the researcher in case they wanted more clarification.

### 3.4. Data analysis

Structural equation modeling (SEM) is a multivariate analysis technique used to estimate various relationships among observed variables and latent variables (Hoyle, 1995). A full SEM model or latent variable model consists of two parts: the measurement model and the structural model. The measurement model relates measured variables to latent variables through confirmatory factor analysis models (CFA models), whereas the structural model links latent variables to one another, referred to as causal modeling or path analysis (Yu and Shek, 2014). For this study, we estimate the structural equation model (SEM) using the maximum likelihood (ML) method, and mainly use the path analysis method to analyze users’ perception of travel intention after browsing short tourism videos through path plots and effect size. IBM SPSS Statistics 26.0 and IBM SPSS Amos 26.0 were used to test the relationships between short video factors and tourism intention, including the process of full latent variable model testing that includes both CFA and path analysis.

### 3.5. Study variables

#### 3.5.1. Telepresence

Minsky (1980) first proposed the concept of telepresence, which refers to the experience of presence that people have in a virtual environment rather than a real environment. Some researchers have defined telepresence as an individual’s perception of the environment, describing the degree of reality that people perceive in the virtual environment, also known as “immersiveness” (Lombard and Jones, 2007; Yao and Jia, 2021; Zheng et al., 2022). Current studies reveal that telepresence affects users’ intention to purchase from shopping websites or to view live broadcasts. In different online situations, telepresence will significantly influence the flow experience of online users (Pelet et al., 2017; Kim and Ko, 2019).

Short tourism videos can create a better experience for users to participate interactively in a strongly telepresent setting (An et al., 2021). When users browse videos with a strong telepresence, they will feel that they are in the midst of mountains and rivers, resulting in a sense of freedom and relaxation throughout the body (Taylor et al., 1998; Green and Brock, 2000; Chaulagain et al., 2019; Chi et al., 2020). Thus, telepresence is the feeling of being in a virtual environment: when users watch short videos, they will feel the scenery in front of them. Flow is an exciting and satisfying experience for users: when users watch short videos, they will feel very happy. A strong telepresence can encourage users to immerse themselves in the videos they browse and yearn for the scenes they depict, thus generating tourism intention. When users feel happy, relaxed and satisfied, telepresence has a positive influence on flow experience.

#### 3.5.2. Flow experience

The concept of the flow experience originates from flow theory as proposed by psychologist Csikszentmihalyi (1975), who defined flow as a feeling of fully investing one’s mental power in a certain activity. When flow occurs, people will be highly excited and satisfied. With the rapid development of the Internet, flow theory has seen wide use in research on consumers’ online activities, with the flow experience invoked to explain their online behaviors (Koufaris, 2002; Valenzuela et al., 2009). Previous researches showed that there was a significant positive correlation between flow experience and intention (Ilhami, 2021; Yao and Jia, 2021; Zheng et al., 2022). Once consumers enter the flow experience, they will be fully invested and even forget time and space in this highly concentrated state. The resulting pleasant emotion will stimulate consumers’ purchase intention (Gong et al., 2019).

When browsing a short tourism video, users will be continually stimulated by the scenery and characters in the video. Spectacular scenery and rich scene switching make it easier to stimulate the enjoyment of the audience and make them enter a committed flow experience (Collins et al., 2009). Moreover, users’ high satisfaction or high expectations can make it easier to enter a flow experience (Hwang et al., 2011). When users browse short tourism videos and enter the flow state, they will unconsciously yearn for the tourism destination in the video, thus generating tourism intention.

#### 3.5.3. Characteristic variables of short videos

The previous research on short video content mainly involves enjoyment, professionalism and interactivity (Choi et al., 2021).

Perceived enjoyment, also known as perceived pleasure, is an internal motivation with a significant impact on users’ engaging in a certain activity; it can also be used to measure the change of flow (Wilder et al., 1989). It is the degree of interest perceived by users when using a specific system for online activities. Some research has confirmed that the interest and entertainment value of a marketing platform’s content have a positive impact on the emotional arousal of users (Beuckels and Hudders, 2016; Xue, 2017). When browsing a short tourism video, if the user thinks the content is very interesting, the perceived enjoyment will promote their immersion in it and have a positive impact on telepresence. Interesting and entertaining short video content arouses users’ emotions, and the perceived enjoyment will positively affect flow experience.

Content professionalism reflects the knowledge, experience and ability of video creators in a certain field and affects the evaluation and perception of users (Li et al., 2022). When studying a popular science vlog, some researchers found that professional and authentic short video content can enable the audience to receive information to the greatest extent in a limited time (Zeng and Lu, 2021; Ma and Zhang, 2022). Therefore, when the perceived professionalism of short video content meets users’ psychological expectations, it will generate the desire to be in the video environment. Perceived professionalism has a positive impact on telepresence. Convincing and professional short video content will stimulate users’ interest, and content professionalism will generate a positive flow experience.

Perceived interactivity refers to the communication and interaction between short video users (Thorson and Rodgers, 2006; Zhao and Lu, 2010). Short video platforms provide channels for likes, forwarding and sharing, and users can leave messages in the comment area to participate in discussions or overlay real-time comments. High perceived interactivity means that users are willing to actively participate in the interaction, obtain the required information and complete the necessary work, which can deepen users’ understanding and use of the short video content (Moon and Kim, 2001; Choi et al., 2021). Attractiveness and interactivity of short video platforms enhance users’ perceived interactivity and have a positive effect on telepresence. Perception of the utilitarian value embodied in interaction and the degree of user participation both have a positive impact on the flow experience (Wu et al., 2014; Zhou et al., 2022).

#### 3.5.4. Perceived usefulness and perceived ease of use

In TAM, perceived usefulness and perceived ease of use significantly affect the intention of using technology (Hansen et al., 2018; Yahia et al., 2018). When users believe that new technologies are useful and easy to use, it produces a certain degree of perceived pleasure, and they are more inclined to use those technologies (Hyowon et al., 2022). Research on mobile short video software has found that these variables have the same relationship (Yan, 2021; Wang et al., 2022).

In this study, perceived usefulness refers to users’ perception that short tourism videos are not only interesting, but also useful for individuals to obtain information, learn about tourism options and improve cultural literacy (Lee and Lehto, 2013). Users are willing to be in the environment of short videos, and their perceived usefulness has a positive impact on telepresence. Users experience a certain degree of life happiness and satisfaction, so perceived usefulness has a positive impact on flow experience.

Perceived ease of use answers the question of how convenient it is to use short video apps or platforms. As mobile media that integrate editing, sorting, sharing and viewing, short video apps have simple operation and strong functionality (Shang and Liu, 2021). Smooth and instinctive operation can help users enter a state of high telepresence, and perceived ease of use has a positive impact on telepresence. When a short video platform is easy to operate, it helps users enjoy the use process and positively affects the flow experience.

## 4. Results

### 4.1. Sample characteristic analysis

Of the 395 participants who submitted a valid questionnaire, 186 were male (47.1%) and 209 were female (52.9%). Among them, the users of tourism short videos are mainly people under the age of 30, who accounted for 69.5% of our valid respondents; 22.1% were in the age group 30–50, and the remaining 8.4% were over 55. 80.6% of users said they like traveling, while only 6.3% of users said they do not. In terms of video browsing frequency, 28.9% of users often browse tourism short videos, 35.1% do so occasionally, 31.7% rarely, and only 4.3% never browse them. Most short video lovers browse tourism scenery (78.6%) and tourism strategies (82.6%), while fewer browse tourism history stories (49.1%) and other categories (19.4%). Most spend 5 to 30 min (69.4%) in a single browsing session, but some users indulge in short video for a long time (23.7%).

### 4.2. Reliability and validity analysis

The reliability of the scale is determined by the internal consistency coefficient (Cronbach’s α), average variance extracted (AVE) and composite reliability (CR). As shown in Table 2, Cronbach’s α of perceived enjoyment, perceived professionalism, perceived usefulness, perceived ease of use, perceived interactivity, telepresence, flow experience and tourism intention are greater than 0.8, the AVE of the factor load value of each measurement item is greater than 0.5, and the CR of each combination reliability is greater than 0.7, indicating that each measurement item of the questionnaire has very good reliability. Validity testing includes content validity and structural validity. After literature research, expert interview and pre-investigation, the design of this research scale extracts and modifies the items. The process is rigorous and has good content validity. The KMO is 0.982, greater than 0.8, the Bartlett’s sphericity value is 11,282.491 (DF = 465), and the statistical significance (P) is less than 0.001, indicating that the research data has high correlation and is suitable for factor analysis. In confirmatory factor analysis (CFA), the factor load value of each item is greater than 0.7, indicating that the validity of the measurement model is good.

**Table 2:** Reliability and validity analysis results of questionnaire.

Dimension	Item	Factor loadings	SMC	CR	AVE	Cronbach’s α
Perceived enjoyment	PE1	0.791	0.626	0.8575	0.6675	0.856
	PE2	0.847	0.717			
	PE3	0.812	0.659			
Perceived professionalism	PP1	0.781	0.610	0.8513	0.5888	0.850
	PP2	0.751	0.564			
	PP3	0.772	0.596			
	PP4	0.765	0.586			
Perceived usefulness	PU1	0.821	0.674	0.8697	0.6255	0.869
	PU2	0.809	0.654			
	PU3	0.770	0.593			
	PU4	0.762	0.580			
Perceived ease of use	PEOU1	0.796	0.634	0.8802	0.6475	0.879
	PEOU2	0.833	0.695			
	PEOU3	0.799	0.638			
	PEOU4	0.790	0.624			
Perceived interactivity	PI1	0.762	0.581	0.8149	0.5249	0.815
	PI2	0.700	0.490			
	PI3	0.666	0.444			
	PI4	0.765	0.586			
Telepresence	P1	0.849	0.721	0.9014	0.6957	0.902
	P2	0.840	0.706			
	P3	0.824	0.680			
	P4	0.823	0.678			
Flow experience	FL1	0.823	0.677	0.8716	0.6937	0.872
	FL2	0.816	0.666			
	FL3	0.859	0.737			
Tourism intention	TI1	0.819	0.672	0.9120	0.6748	0.912
	TI2	0.783	0.614			
	TI3	0.801	0.641			
	TI4	0.848	0.719			
	TI5	0.854	0.729			

It can be seen from the above Table 3 that the absolute values of skewness coefficient and kurtosis coefficient are less than 1.96, which can be considered that this group of sample data conforms to the normal distribution and is suitable for the structural equation model method.

**Table 3:** Test of normal distribution of questionnaire.

Dimension	Items	Average	Standard deviation	Skewness	Kurtosis
perceived enjoyment (PE)	PE1	3.52	1.222	−0.612	−0.440
	PE2	3.58	1.207	−0.767	−0.317
	PE3	3.58	1.207	−0.614	−0.552
Perceived professionalism (PP)	PP1	3.55	1.302	−0.644	−0.676
	PP2	3.45	1.230	−0.532	−0.579
	PP3	3.63	1.186	−0.764	−0.231
	PP4	3.50	1.132	−0.515	−0.521
Perceived usefulness (PU)	PU1	3.55	1.274	−0.674	−0.583
	PU2	3.64	1.297	−0.647	−0.696
	PU3	3.61	1.184	−0.709	−0.308
	PU4	3.68	1.261	−0.684	−0.548
Perceived ease of use (PEOU)	PEOU1	3.63	1.232	−0.642	−0.596
	PEOU2	3.64	1.229	−0.694	−0.527
	PEOU3	3.55	1.108	−0.700	−0.268
	PEOU4	3.60	1.172	−0.656	−0.363
Perceived interactivity (PI)	PI1	3.49	1.150	−0.416	−0.594
	PI2	3.56	1.189	−0.621	−0.437
	PI3	3.48	1.293	−0.546	−0.658
	PI4	3.54	1.207	−0.571	−0.519
Telepresence (TP)	TP1	3.62	1.250	−0.689	−0.522
	TP2	3.43	1.216	−0.570	−0.594
	TP3	3.59	1.252	−0.663	−0.541
	TP4	3.56	1.223	−0.644	−0.460
Flow experience (FL)	FL1	3.71	1.297	−0.685	−0.602
	FL2	3.63	1.144	−0.803	−0.094
	FL3	3.54	1.205	−0.704	−0.420
Tourism intention (TI)	TI1	3.61	1.221	−0.696	−0.412
	TI2	3.59	1.131	−0.589	−0.396
	TI3	3.68	1.151	−0.740	−0.256
	TI4	3.63	1.308	−0.709	−0.613
	TI5	3.75	1.300	−0.751	−0.678

### 4.3. Structural model

Amos26.0 is used to study the overall fitting evaluation and hypothesis test of the model. It can be seen from Table 4 that the model fitness meets the standard, indicating that the data collected and the model constructed match well, the proposed path assumption relationship is consistent with the actual situation, and the model coefficient results are accurate and effective.

**Table 4:** Model fitness parameters.

Fitting coefficient	Evaluation criterion		Actual value	Fitting situation
	Good	Acceptable		
X2/DF	< 3	3.0–5.0	2.060	Good
GFI	> 0.9	0.7–0.9	0.884	Acceptable
AGFI	> 0.9	0.7–0.9	0.860	Acceptable
CFI	> 0.9	0.7–0.9	0.961	Good
RMR	Close to 0	< 0.5	0.037	Acceptable
TLI	> 0.9	0.7–0.9	0.956	Good
RMSEA	< 0.08	0.08–0.1	0.052	Good

**Figure 2:**
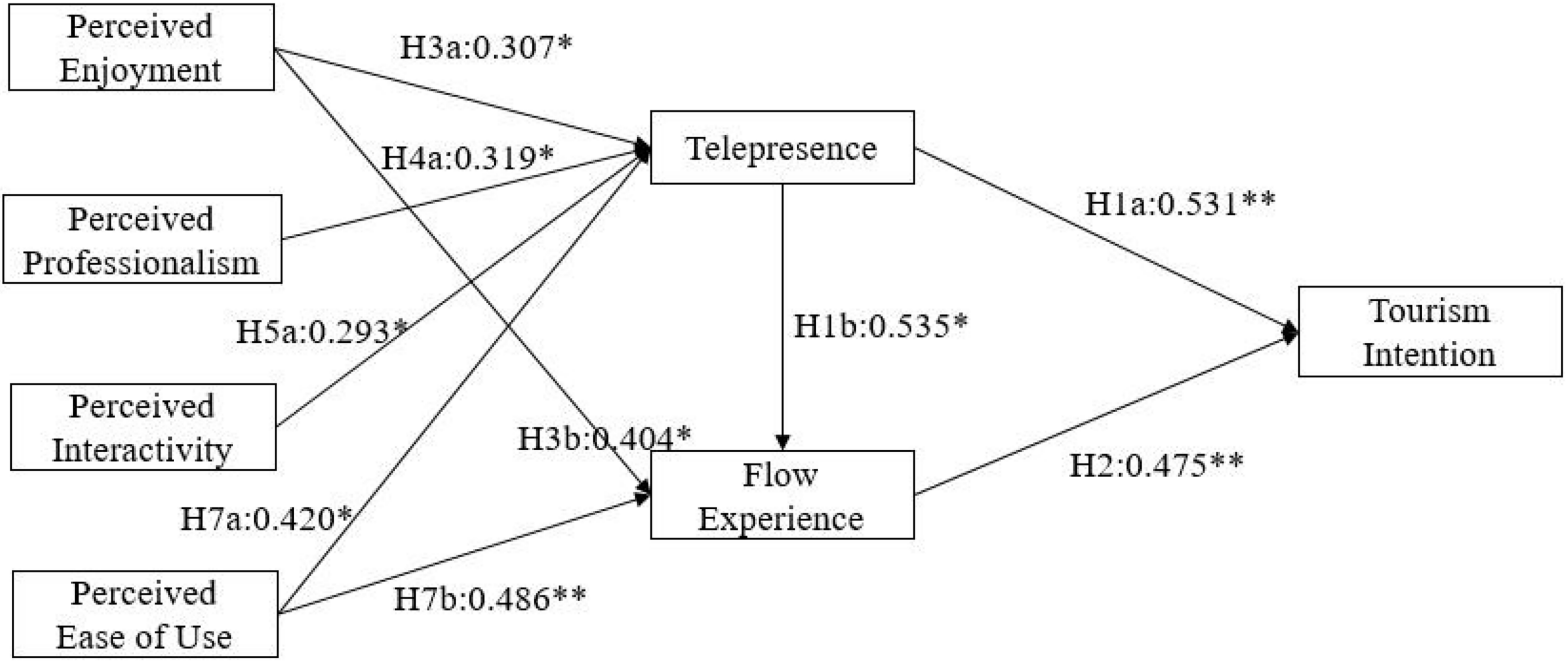
Model path verification. *p < 0.05; **p < 0.01.

The degree of interpretation of the whole model and the significance of relevant assumptions are evaluated by path coefficient, C.R. value and *p* value, as shown in Table 5. The absolute value of the critical ratio C.R. of 9 paths is greater than 1.96, and the significance probability value *p* is less than 0.05; these hypotheses are accepted. The absolute value of the critical ratio C.R. of 4 paths is less than 1.96, and the significance probability value p is greater than 0.05; these hypotheses are rejected (see Figure 2). The verification results in Table 4 show that some hypotheses proposed in this paper have passed the test.

**Table 5:** Analysis of model results.

Hypothesis	Path	Unstandardized regressive coefficient	S.E.	C.R.	*P*	Standardized regressive coefficient	Result
H1a	TI ← TP	0.520	0.178	2.921	0.003	0.531	Accepted
H1b	FL ← TP	0.542	0.246	2.204	0.028	0.535	Accepted
H2	TI ← FL	0.460	0.177	2.598	0.009	0.475	Accepted
H3a	TP ← PE	0.320	0.161	1.985	0.047	0.307	Accepted
H3b	FL ← PE	0.426	0.201	2.124	0.034	0.404	Accepted
H4a	TP ← PP	0.356	0.174	2.045	0.041	0.319	Accepted
H4b	FL ← PP	−0.129	0.199	−0.647	0.518	−0.114	Rejected
H5a	TP ← PI	0.348	0.174	1.997	0.046	0.293	Accepted
H5b	FL ← PI	−0.194	0.176	−1.104	0.270	−0.162	Rejected
H6a	TP ← PU	−0.392	0.231	−1.700	0.089	−0.350	Rejected
H6b	FL ← PU	−0.167	0.222	−0.753	0.452	−0.148	Rejected
H7a	TP ← PEOU	0.485	0.234	2.075	0.038	0.420	Accepted
H7b	FL ← PEOU	0.568	0.193	2.948	0.003	0.486	Accepted

*p < 0.05; **p < 0 .01; ***p < 0.001.

## 5. Discussion

The aim of the present study is to investigate the influence of short video factors (perceived enjoyment, perceived professionalism, perceived interactivity, perceived ease of use, perceived usefulness) on tourism intention by extending the SOR model with these factors. The findings revealed that perceived enjoyment and perceived ease of use are significant determinants of both telepresence and flow experience, which in turn positively associate with users’ tourism intention. This implies that the expectations for perceived enjoyment and perceived ease of use are significantly correlated with telepresence and flow experience in browsing short tourism videos. These results support the findings of a recent preliminary study which revealed that the convenience of a short video app or platform can significantly improve users’ sense of experience when browsing short videos, and smooth, instinctive operation can help them enter a state of high telepresence and strong flow experience faster (Wang et al., 2022). These findings also reconfirm the conclusions of many previous researchers (Beuckels and Hudders, 2016; Xue, 2017; Hyowon et al., 2022). Meanwhile, Choi et al. (2021) finds that the perceived enjoyment impacts of short video itself play a great auxiliary role in making it pleasant and interesting to browse short videos.

At the same time, the results show that perceived professionalism and perceived interactivity have a significant positive impact on telepresence, consistent with prior studies that found these same factors to have a positive impact on people’s intention to use websites (Stavropoulos et al., 2013; Wu et al., 2014; Beuckels and Hudders, 2016; Zhou et al., 2022). Ma and Zhang (2022) believed that users tend to judge professionalism of video content in editing, special effects, narration, and copywriting, and that users’ recognition of high professionalism can quickly immerse them in short tourism videos. Interactivity is also reflected in overlaid real-time comments (“bullet screens”) on short videos, in the exchange and discussion in the comment area, and in likes, forwarding and sharing. Users’ active participation in videos can quickly immerse them in short tourism videos, thus generating telepresence and ultimately improving users’ tourism intention (Choi et al., 2021).

Although most relevant studies show that users’ perceived usefulness of new technologies or systems will generate flow experience and telepresence in some settings (Hansen et al., 2018; Yahia et al., 2018; Yan, 2021), the results show that this relationship does not necessarily apply to all people in any case. One possible reason is that the content of tourism short videos is commonplace among short video lovers, and they are no longer easily immersed in it. Another is that the relationship between perceived usefulness and telepresence and flow experience may depend on the motivation judgment of short video users (Mohsin et al., 2017; Bayih and Singh, 2020). Additionally, the results revealed that a feature in the short video is more attractive or important, even if the content of the short video is considered useless, the user will still have a sense of flow and telepresence when watching the short video (Skard et al., 2021). At the same time, since the samples of this study are concentrated on short video lovers, this relationship may not be established due to sample deviation.

Moreover, the results revealed that telepresence not only has a direct and significant positive impact on tourism intention, but also has an indirect impact on flow experience through significant prediction. The telepresence-intention link has previously been confirmed in consumer behavior research (Pelet et al., 2017; Kim and Ko, 2019; Ye et al., 2020), and its reconfirmation in tourist behavior research further demonstrates the robustness of this association. Zheng et al. (2022) believed that telepresence is a state in which users can spontaneously be in the immersive state when browsing short videos. It is an important embodiment of users’ yearning for tourism destinations. This implies that when users immerse themselves in it, they will naturally forget the passing time and generate a sense of pleasure to a certain extent. That is, they enter the flow state. In both cases, the act of watching such videos will arouse users’ inner desire for tourism and generate tourism intention.

## 6. Conclusion

With the development of science and technology, new technologies such as 5G and virtual reality have entered people’s daily life. Short tourism videos have already had an impact on users’ tourism intentions, and new media and technologies have promoted the development of tourism in China and around the world. This research shows that the operation of short video platforms and users’ short video experiences have a significant relationship with users’ tourism intention. Short video platforms provide convenient operation, easy communication and sharing, and high-quality services to promote the tourism intentions of Chinese users. The findings show that short video characteristics and perceived ease of use have a significant impact on telepresence and flow experience, and thus have a significant impact on tourism intention. This study is noteworthy because short video has become an important way for most prospective visitors to understand tourist attractions in the post-pandemic era. Promoting the development of short tourism videos will be the key path to improve users’ tourism intention.

## 7. Theoretical and practical implications

This study holds several theoretical implications. First, the developed conceptual model is considered the first to combine short video factors and technology acceptance factors to extend the SOR model to predict users’ tourism intention. Second, the constructed model explains the changes in users’ tourism intention in the context of the global short video boom. Third, most of the established paths in the developed model are statistically positive. Thus, it is assumed that the short video factors and the SOR model are matched, which is one of the contributions of this study. Fourth, considering that few studies have extended the SOR model to address short video, future researchers may use this model to explore the impact of short video factors in other settings. Fifth, the outcomes of this study enhance the understanding of the key role that short video factors play in improving users’ tourism intention.

There are also several practical implications of this study. First, the outcomes of this study will support developers and practitioners in considering technology acceptance factors while designing and developing short video platforms for online social media companies. Second, the marketing departments of tourism destinations may also use the results of this study in designing and creating short videos by combining the factors of short video content construction to enrich their publicity channels. Third, since perceived usefulness was found not to support users’ tourism intention, short video producers need to pay more attention to the creation of short video content to obtain greater attention and promote better flow.

## 8. Limitations and future recommendations

This work may also be subject to some limitations. First, more factors that influence users’ tourism intention, such as celebrity effect, social environment, etc., need to be addressed to comprehensively examine the factors by which short videos influence users’ tourism intention. Second, while the respondents in this study are all Chinese, short video platforms such as TikTok and Kwai are already in the process of globalization. It is thus worth studying whether different cultural backgrounds will affect user experience after browsing short tourism videos, which necessitates a future program of cross-country multi-sample research. Third, this study employs stratified sampling in selecting the respondents, which in turn may hinder the generalizability of the results. Thus, further attempts could consider other sampling techniques. Fourth, the short video industry is still making breakthroughs and innovations: VR, AR, and other technologies are being integrated, all of which require further theoretical research.

## Data availability statement

The raw data supporting the conclusions of this article will be made available by the authors, without undue reservation.

## Ethics statement

In accordance with the local legislation and institutional requirements, the questionnaire can only be carried out after fully explaining to the participants, after their agreement they begin to answer the questions. The whole survey process and results will not involve the disclosure of the participants’ personal identity information.

## Author contributions

JL and YW: conceptualization. JL: methodology and project administration. YW: software, investigation, and writing—original draft preparation. LC: validation and writing—review and editing. All authors have read and agreed to the published version of the manuscript.

## Conflict of interest

The authors declare that the research was conducted in the absence of any commercial or financial relationships that could be construed as a potential conflict of interest.

## Publisher’s note

All claims expressed in this article are solely those of the authors and do not necessarily represent those of their affiliated organizations, or those of the publisher, the editors and the reviewers. Any product that may be evaluated in this article, or claim that may be made by its manufacturer, is not guaranteed or endorsed by the publisher.
